# The mechanism of Co oxyhydroxide nano-islands deposited on a Pt surface to promote the oxygen reduction reaction at the cathode of fuel cells†

**DOI:** 10.1039/d0ra08645b

**Published:** 2020-12-17

**Authors:** Jinghao Lu, Libin Yang, Wei Guo, Songtao Xiao, Lingyu Wang, Yinggen OuYang, Peng Gao

**Affiliations:** College of Chemical Engineering and Materials Science, Tianjin Key Laboratory of Brine Chemical Engineering and Resource Eco-utilization, Tianjin University of Science and Technology China yanglibin@tust.edu.cn +86-13752339079; Department of Radiochemistry, China Institute of Atomic Energy Beijing 102413 China

## Abstract

With the rapid development of fuel cell technology, the low reduction rate of oxygen on Pt-based cathodes is generally considered the main obstacle. Pt/transition metal alloys (Pt–Ms) or Pt/transition metal oxides (Pt–MO_*x*_) can be formed by doping transition metal atoms into the lattice of the Pt layer or depositing onto the surface of the Pt layer to intensify the catalytic activity of the electrodes. In this work, a stepwise solution chemical reduction method for high dispersion of cobalt oxyhydroxide (–OCoOH) deposited onto the facet of Pt as nano-islands and the mechanism of promoting the oxygen reduction reaction (ORR) at the cathode have been investigated by density functional theory (DFT) calculation. As a result, the electrocatalytic activity of Pt with nano-island –OCoOH structure was 3.6 times that of the Pt/C catalyst, which indicated that promoting the desorption of the first O atom and weakening the adsorption capacity of the interfacial junction Pt for the second O atom from adsorbed oxygen attributed to the migration of d-band center in Pt and the existence of the Co hydroxyl group.

## Abstract 2

The Pt–OCoOH structure has a positive effect on the ORR; the bond length, binding energy and d-band center were investigated to explain the improved ORR activity.

## Graphical abstract

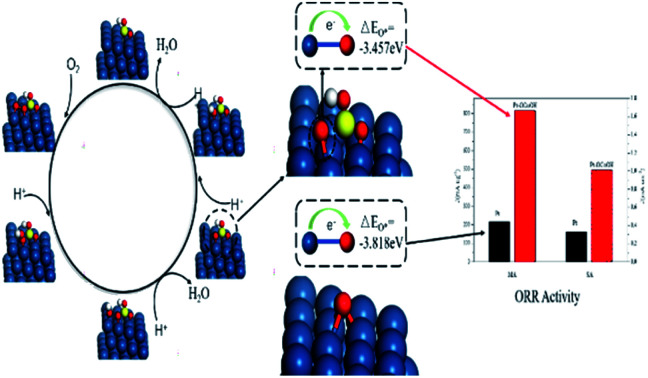

## Introduction

Nowadays, with the over-exploitation of fossil fuels, environmental issues have become the biggest challenges facing mankind. Fuel cells are being considered as new energy conversion devices as they have high energy conversion efficiency without the Carnot cycle restrictions and are environmentally friendly.^1–5^ Electrode catalyst materials are one of the core components of a fuel cell, and are key indicators of electrocatalytic activity and fuel cell performance. The noble metal Pt is usually used as an active substance of the catalyst in proton exchange membrane fuel cells (PEMFCs), but the disadvantages of low Pt reserves and susceptibility to poisoning has restricted the commercial development of the fuel cell.^6–8^ Pt is currently the most active catalyst in fuel cells. However, the rate of oxygen reduction reaction (ORR) with Pt as cathode catalytic active center is much lower than that of anodic oxidation reaction. The oxidation and reduction rates at the two electrodes do not match, significantly limiting the application of Pt in fuel cells as electrode catalyst materials. Thus, the low ORR activity of platinum as the cathode, caused by the strong adsorption for oxygen species, is one of the key obstacles to developing and promoting platinum-based electrode materials for fuel cells.^9^

Although the strong adsorption of oxygen molecules is favourable for ORR in the initial stage, it greatly limits the oxygen species desorption, and the adsorption of OH^−^ on the Pt surface reduces the number of active sites to give bare Pt, also affecting the ORR activity at the cathode.^10–12^ The electronic structure and d-band center position of the Pt electrode are commonly used to describe and explain the electrocatalytic activity.^13–15^

Previous studies have done comprehensive research to improve the ORR activity of the cathodes of fuel cells. In summary, the studies suggested the following three solutions. First, reducing the size of Pt clusters and providing more active sites can improve the utilization of the precious metal Pt.^16–18^ Second, transition metals such as Co, Ni, and Fe introduced into the Pt lattice to form alloys can improve the activity of Pt catalysts as the synergistic effect of two kinds of metals changes the electronic and chemical properties.^1,19–24^ Third, platinum-free non-precious metals have been developed and applied, such as N-doped carbon nanotubes^25^ and Fe–N–C catalyst.^26–28^ The introduction of transition metal elements has been widely and systematically studied. It was found that the reduction of the d-band center of Pt and the adsorption capacity of Pt for O enhances the activity of Pt for ORR. However, with the gradual precipitation of transition metals, the deactivation of Pt–M (Fe, Co, Ni) alloy can occur, which will restrict their applications as electrode materials. Liu *et al.*^29^ used near-ambient pressure scanning tunneling microscopy (NAP-STM) to reveal the phase transition of the surface morphology of Pt_3_Co in the atmosphere of O_2_ and hydroxide of Co was formed on the surface of Pt but their study did not give the evidence and results on the deactivation of the hydroxylated alloy. R. Soni *et al.*^30^ then proved that the process of Co segregation to the surface to form Co oxide may not be the main reason for the deactivation of the Pt–M bimetallic catalyst by density functional theory (DFT) calculation.

Cobalt, in this work, as a late transition metal with unfilled 3d orbital, was hydroxylated and deposited on the surface of the Pt atomic layer to form a stable and highly dispersed Pt–OCoOH by the stepwise solution chemical reduction method under alkaline conditions. The activity and stability of Pt–OCoOH/C as the cathode for ORR was demonstrated by electrochemical performance tests. The electronic structure and energetics of Pt–OCoOH reveal the mechanism for the enhancement of ORR activity by quantum chemical calculation based on DFT.

## Experimental section

### Materials and chemicals

Cobalt nitrate trihydrate (Co(NO_3_)_2_·6H_2_O), chloroplatinic acid (H_2_PtCl_6_·6H_2_O), urea (CH_4_N_2_O), and ethylene glycol (EG) were purchased from Aladdin; sodium hydroxide (NaOH), potassium hydroxide (KOH) and trisodium citrate dihydrate (Na_3_C_6_H_5_O_7_·2H_2_O) were offered by Aladdin Chemical Reagent (Shanghai) Co., Ltd. All the chemicals were of analytical grade and used as received. Throughout the experiment, all aqueous solutions were prepared with distilled water.

### Preparation of Pt–OCoOH

**Scheme 1:**
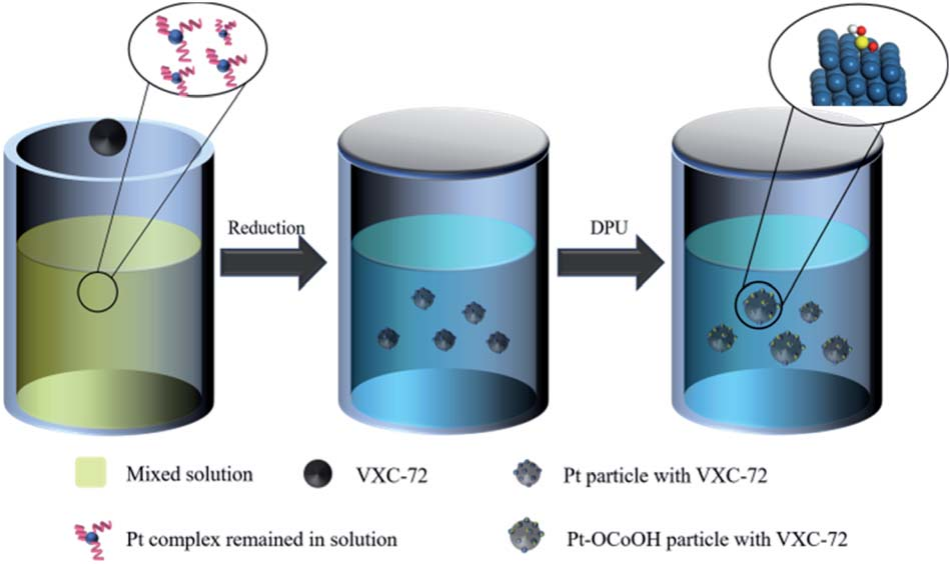
Illustration of the synthesis of Pt/VXC-72 electrocatalysts.

Typically, the procedure of Pt–OCoOH/C synthesis was schematically divided into two steps (as shown in Scheme 1). Nano-precious metal Pt particles were prepared by liquid phase reduction with H_2_PtCl_6_ solution. Then, Pt–OCoOH was successfully prepared by the precipitation and impregnation of nano Pt particles with urea (DPU) as the sustained release agent.^31^ The preparation procedure is described below.

First, 66.83 mg of H_2_PtCl_6_·6H_2_O (0.13 mmol) was thoroughly mixed with 0.010 g trisodium citrate dihydrate (Na_3_C_6_H_5_O_7_·2H_2_O) in EG (100 mL), and the pH was adjusted to 11 by using NaOH solution. Then, 100 mg of carbon (Vulcan XC-72) was added to the uniform solution (the weight ratio of Pt : C = 20%). The mixture was subjected to centrifugation at 12 000 rpm for 20 min, followed by thorough washing with water and ethyl alcohol, and finally drying at 60 °C in vacuum for standby applications. Then, 25 mg of the prepared Pt particles adsorbed on C was slowly added to 100 mL of aqueous solution containing 0.072 mg Co(NO_3_)_2_·6H_2_O and 4.5 g urea. The hydroxyl groups released by the continuous decomposition of urea slowly caused precipitation on Pt particles. After heating in an oil bath at 90 °C for 24 hours, the precipitate was separated by centrifugation at 12 000 rpm, washed repeatedly with deionized water and ethanol, and then placed in an oven to dry overnight at 80 °C.

### Characterization and electrochemical performance tests

The crystalline phases were determined by using an X-ray powder diffraction (XRD) instrument operated at 36 kV, 30 mA using Cu Kα X-rays (*λ* = 0.15405 nm). The morphology of the samples was tested by transmission electron microscopy (TEM) and energy-dispersive X-ray spectroscopy (EDS) on an APREO transmission electron microscope at an accelerating voltage of 200 kV. X-ray photoelectron spectroscopy was performed with a Thermo 250Xi spectrometer for determining the valence of Pt and Co atoms. C 1s peak was used as the reference, which was at 284.8 eV.

All electrochemical characteristic measurements were carried out on a CHI 760E electrochemical analyzer (Shanghai Chen Hua Instrument Co. Ltd.). The electrochemical performance tests were performed in a traditional three-electrode cell system with a modified glassy carbon electrode (GCE, 3 mm in diameter) as the working electrode and a carbon rod as the counter electrode, and the reference electrode was a saturated silver–silver chloride (Ag/AgCl) electrode. Herein, all the potentials were measured against a reversible hydrogen electrode (RHE).

The suspension ink was made by mixing 2.0 mg of the as-prepared final catalyst in 1 mL of water and ethanol mixture with 20 μL of 5% wt. Nafion solution was prepared by ultrasonication for 30 min. Details of the catalyst dosage are provided in the ESI, Table S1.† 3 μL of the suspension (2 mg mL^−1^) was dropped onto the electrode surface and then dried in air. In addition, as-prepared nano-particle Pt was prepared as ink and coated on the electrodes by the same way for comparison. The cyclic voltammetry (CV) curves of the catalysts were obtained in N_2_ saturated 0.5 mol L^−1^ H_2_SO_4_ solution at a scan rate of 50 mV s^−1^. The electrocatalytic activity of the as-prepared catalysts for ORR was evaluated in an oxygen saturated KOH solution (0.1 mol L^−1^) with linear sweep voltammetry (LSV) at a scan rate of 10 mV s^−1^ with rotation rates ranging from 400 to 2500 rpm under O_2_ or N_2_ saturation using a rotating disk electrode. Furthermore, the stability of the as-prepared catalyst was determined in 0.1 mol L^−1^ KOH solution saturated with N_2_ or O_2_ at a scan rate of 100 mV s^−1^ from 0.6 V to 1.0 V for 10 000 cycles.

## Computational details

The calculations for the electronic structure and adsorption energy of Pt–OCoOH were performed with the Vienna *Ab initio* Simulation Package (VASP)^32–35^ based on density functional theory (DFT).

The close-packed facet of Pt (111) was modeled by a 4-layer slab with a 4 × 4 × 1 supercell and separated by a 15 Å vacuum spacer to avoid interaction between the neighbors. The Co, O, and OH groups were placed onto the top layer of this 3-layer slab to form Pt–OCoOH. After structural optimizations, the positions of the Pt atoms in the two top surface layers were relaxed, and the Pt atoms at the bottom layer were fixed.

While modeling, the interactions between valence electrons and ion cores were treated by Blochl's all-electron-like projector augmented wave (PAW) method.^36^ The exchange–correlation functional was obtained by the generalized gradient approximation following the Perdew–Burke–Ernzerhof scheme, known as GGA-PBE. The plan-wave kinetic energy cutoff was 400 eV, and the *K*-point meshes were set as 4 × 4 × 1. All structures were fully relaxed until the convergence in energy, the force of which reached 1.0 × 10^−6^ eV and 1.0 × 10^−2^ eV Å^−1^, respectively. The spin polarization was considered in the model. The adsorption state and adsorption energy of small molecules such as O_2_ were calculated, and the formula of adsorption energy is given below:

1 *E*_ads_ = *E*_group+slab_ − *E*_group_ − *E*_slab_

2
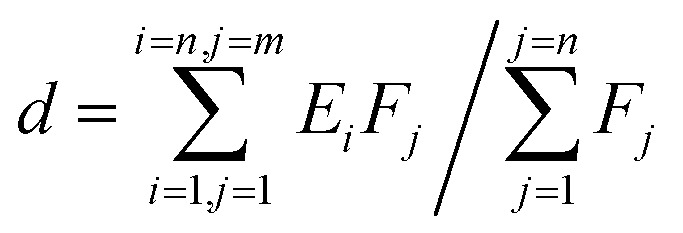
where *E*_ads_ represent the adsorption energy of a specific molecule on the surface of the catalyst, in eV; *E*_group_ represents the total energy of the ultimate stable structure; *E*_slab_ represents the energy of the system after optimization on the (111) facet of Pt excised from the supercell; *E*_group_ represents the energy of a single absorbed molecule in the gas phase. The value of *E*_ads_ calculated according to formula (1) was generally negative, which proves that the energy of the constructed system decreases after adsorption of specific molecules. The adsorption of the induced groups, as a spontaneous process in thermodynamics, is a process of energy reduction. The more negative the value of adsorption energy is, the stronger is the adsorption of the group,^37^ and the more stable the ultimate structure is.

The d-band center of the materials, firstly proposed by Nørskov,^38,39^ was adopted to describe the catalytic activity as the independent descriptor. The value of the d-band center is calculated by formula (2), where *E*_*i*_ represents different energy values and *F*_*j*_ represents the density of states of the d-orbital corresponding to energy values. The more the electron density near the Fermi energy level, the better the catalytic activity.

## Results and discussion

### Characterization of Pt–OCoOH

**Fig. 1:**
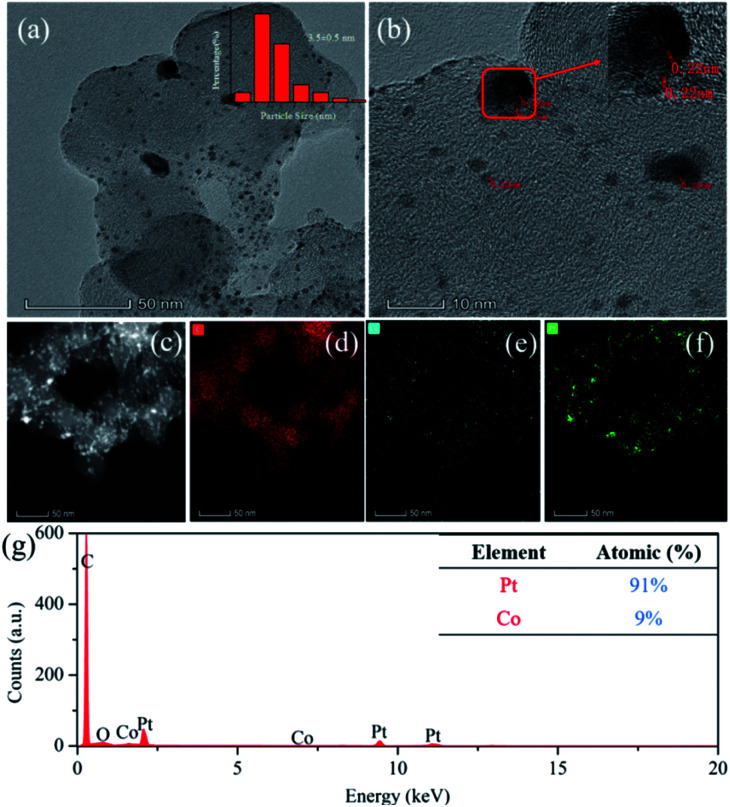
TEM images of (a) Pt–OCoOH/C particle and (b) HR-TEM images showing the Pt size distribution and location. (c–f) High-angle annular dark-field (HAADF)-STEM image and the corresponding EDS elemental mapping of Pt–OCoOH, (d, e and f) represent C, Co, Pt elements, respectively. (g) EDS profile of the Pt–OCoOH/C sample.

As shown in Fig. 1, the nano-particle Pt clusters were evenly distributed on the carbon carrier, with an average particle size of about 3.5 nm (Fig. 1a). In addition, single nanoparticle Pt in Pt–OCoOH nanoparticles had distinct lattice streaks, with a spacing of about 0.22 nm. The presence of alloyed features was verified by STEM-EDS elemental mapping analysis, emphasizing the homogeneous distribution of Pt and Co across the analysed zone (Fig. 1c–f). ICP-OES analysis presented that the content of Pt in Pt/C and Pt–OCoOH/C were 18.8 wt% and 17 wt%, respectively, and EDS spectra (shown in Fig. 1g) indicated that the target elements Pt, Co and O co-existed in the nanomaterials, with the atomic ratio 9 : 1 for Pt to Co.

**Fig. 2:**
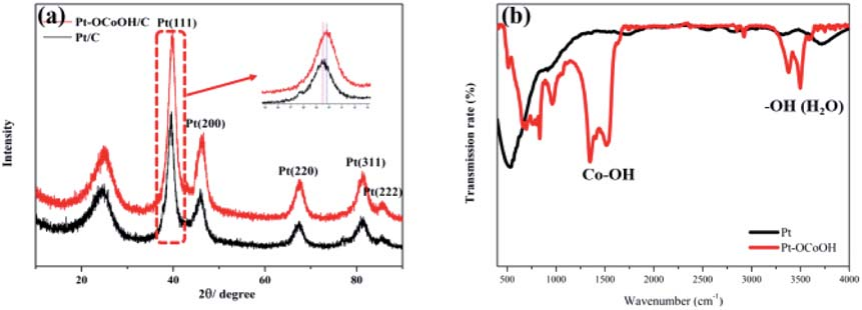
XRD (a) and TT-IR (b) patterns of Pt–OCoOH and Pt/C.

The crystal structures were investigated by XRD analysis. As shown in Fig. 2, the as-prepared catalysts show an obvious face-center-cubic (FCC) structure. A peak at approximately 25° is observed in all samples, and this peak belongs to the C (002) plane.^40^ For Pt/C, the diffraction peaks at 39.76°, 46.24°, 67.45°, 81.28° and 85.71° correspond to the Pt (111), Pt (200), Pt (220), Pt (311) and Pt (222) planes, respectively.^41^ Compared with the standard diffraction patterns of Pt/C, the diffraction peaks for Pt–OCoOH/C shift to smaller angles, which indicates that the presence of Pt–O–Co reduces the distance between Pt–Pt atoms. To further demonstrate the surface structure of as-prepared materials, the FTIR spectra of two kinds of materials (Pt and Pt–OCoOH) were studied and compared. Fig. 2b illustrates that the two distinct twin peaks at 1279 cm^−1^-1484 cm^−1^, assigned to the in-plane bending vibration peaks of Co–OH,^42^ were characteristic to the sample of Pt–OCoOH. However, no obvious peak within the non-fingerprint peak range of 1200–4000 cm^−1^ was observed for pure Pt catalyst.

**Fig. 3:**
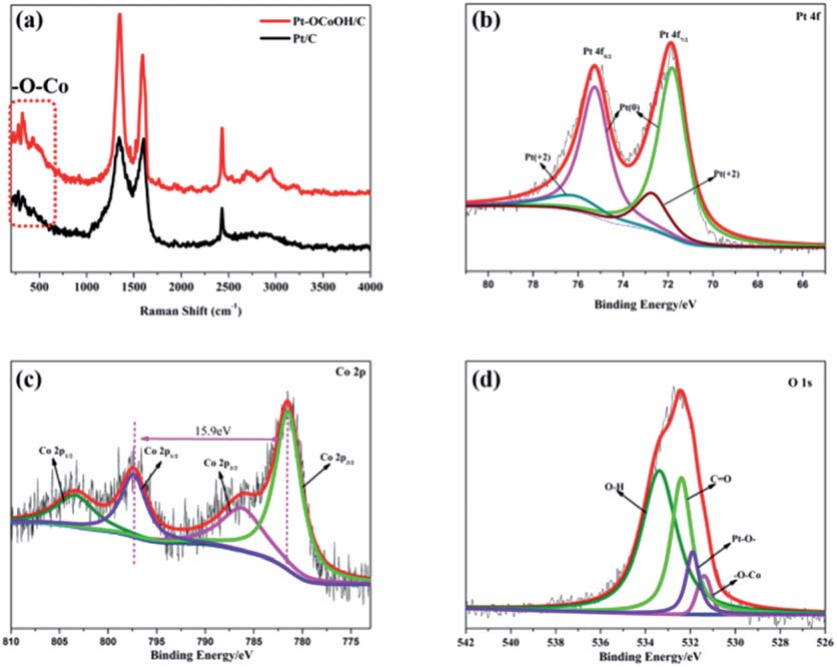
(a) Raman spectra of Pt/C and Pt–OCoOH/C, (b) Pt 4f of the as-prepared nanomaterials with C, (c) Co 2p and (d) O 1s.

To detect the surface state of the as-synthesized photocatalysts, the –OCoOH structure in Pt–OCoOH was studied through Raman and XPS spectroscopy, as shown in Fig. 3a. Compared with pure Pt/C, the Raman shifts showed two special peaks at 350 cm^−1^ and 2850 cm^−1^, which belong to the chemical bond of –O–Co and –OH, respectively.^42^ The XPS spectra show that the four elements Co, O, C and Pt all exist in the prepared catalysts. On the (111) facet of the Pt layer, as shown in Fig. 3b, two strong peaks located at 74.8 and 71.4 belong to Pt 4f_5/2_ and Pt 4f_7/2_ of the zero valence Pt, respectively. The position of the zero valence Pt peaks of the as-prepared Pt–OCoOH was blue-shifted by about 0.4 eV compared with that of the original nanoparticle Pt. Based on the principle of the photoelectric effect, the increase in binding energy between Pt atoms releases the collected electron kinetic energy.^43^ The binding energy of Pt^2+^ at 76.15 eV and 72.70 eV is obviously weaker than that of Pt^0^.

The high-resolution XPS profile of Co shows two broad sets of signals corresponding to 2p_3/2_ (781.35 eV) and 2p_1/2_ (797.25 eV) excluding the presence of metallic cobalt (778.0 eV) at the surface of the Pt–OCoOH.^44^ The signal at 781.25 eV is a satellite peak of Co oxides and hydroxides. More evidence must be collected by analyzing the gap between 2p_1/2_ and 2p_3/2_ peaks and the structure of the satellite bands. At 781.35 eV, 786.45 eV and 802.5 eV, intense satellite peaks were observed, which is a characteristic of high spin Co^2+^ compounds. At the same time, different spin states can be detected from the energy gap between the Co 2p_1/2_ and Co 2p_3/2_. For example, this energy gap was found to be 15.4 eV for low spin Co^2+^ and 16.0 eV for high spin Co^2+^.^45–47^ The energy gap of Pt–OCoOH between Co 2p_1/2_ and Co 2p_3/2_ was 15.9 eV, which suggested the presence of Co with high spin Co^2+^ form. It also suggests that Co is surrounded by Co–OH.^48^ The hydroxide peak in Fig. 3d also confirmed the formation of Co–OH. Besides, the Pt–O band and –OCo band were also found at 531.9 eV and 531.4 eV, respectively. Together, the results demonstrated that hydroxyl cobalt was present on the Pt surface as Pt–OCoOH.

### Electrocatalytic behavior of Pt–OCoOH

The electron transfer number of the catalyst for ORR was calculated by the Koutecky–Levich equation,^49^ using formulas (3) and (4).3
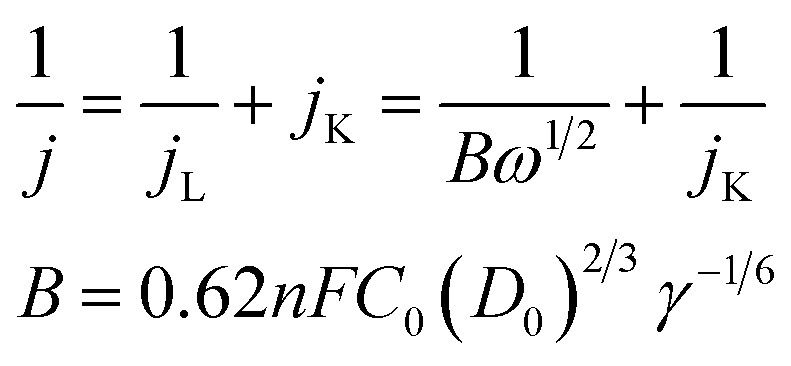
where *j* represents the measured current density; *j*_K_ and *j*_L_ represent the kinetic and diffusion limiting current densities, respectively; *ω* represents the angular velocity of the disk; *n* represents the overall number of electrons transferred in the reaction; *F* represents the Faraday constant (*F* = 96 485 C mol^−1^); *C*_0_ represents the bulk concentration of O_2_ (1.2 × 10^−3^ molL^−1^); *D*_0_ represents the diffusion coefficient of oxygen (1.9 × 10^−5^ cm^2^ s^−1^); *g* represents the kinematic viscosity (*g* = 0.01 cm^2^ s^−1^) of the electrolyte. *i*_k_ can be calculated by the following eqn (4):^50^4
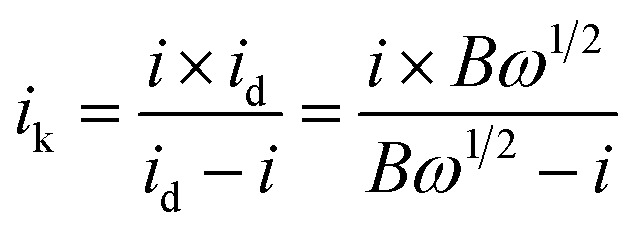


All the measured currents were converted to current density by normalization to the geometric surface area of the modified electrode (*i.e.*, 0.07065 cm^2^ for GCE and RDE). The Pt content was 0.0144 mg cm^−2^ in the catalyst modified GCE.

The specific activity (SA) was obtained from the current density by normalizing the electrochemical active area (ECSA) and Pt content using eqn (5). The mass activity (MA) was estimated by normalizing the content of Pt with eqn (6); in both eqn (5) and (6), *m* represents the content of Pt.^51^5
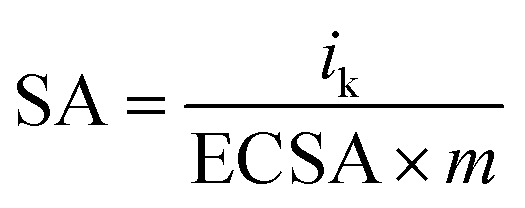
6
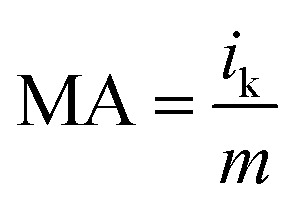


**Fig. 4:**
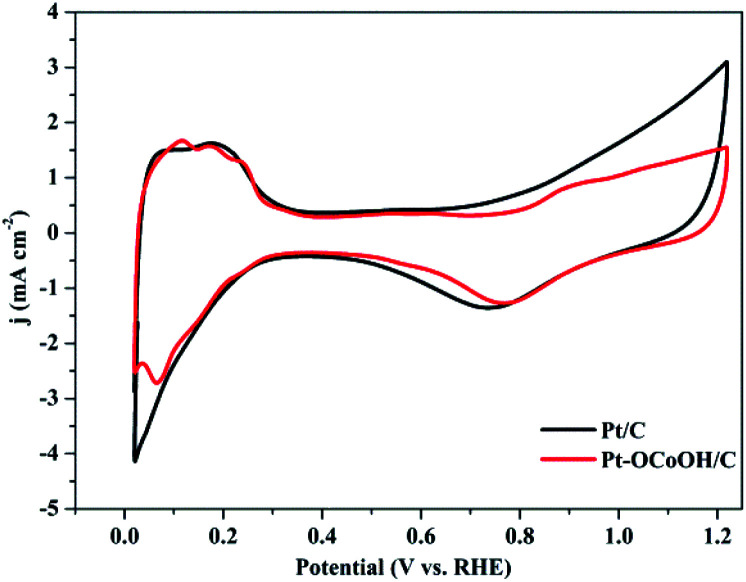
CV curves of Pt–OCoOH and Pt in nitrogen saturated 0.5 mol L^−1^ H_2_SO_4_ solution at a scan rate of 50 mV s^−1^.

The ECSAs of all the as-prepared catalysts, estimated from the hydrogen desorption peak of CV by the H_2_-stripping method in 0.5 M H_2_SO_4_ solution under a N_2_-saturated condition with the potential from 0 to +1.2 V/RHE, are shown in Fig. 4 and the data is summarized in Table 1. There was no significant change in the ECSA of the two self-made catalysts before or after the introduction of –OCoOH, and the as-prepared catalysts presented similar ECSA with other literatures.

**Table 1:** ECSA for different material supported mono/bimetallic catalysts in 0.5 mol L^−1^ H_2_SO_4_

Electrocatalyst	Shape	ECSA (m^2^ g^−1^)	References
PtPd/C	Nanodendrites (NCs)	58.12	^52^
PtNi/C	Octahedral	38.5	^53^
Pt_3_Co/C	Particles	32.9	^54^
Pt–Co/C	Particles	23.24	4
Pt/C	Particles	43.4	This work
Pt–Co(OH)_2_/C	Particles	48.2	This work

**Fig. 5:**
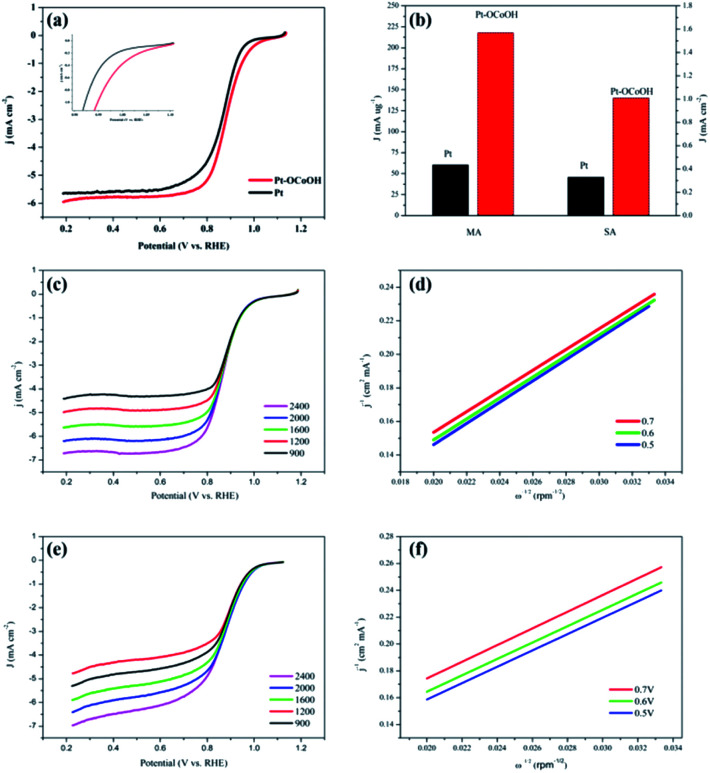
The LSV polarization curves for ORR (a) and the mass ratio activity bar graph, the area ratio activity of Pt/C and Pt–OCoOH/C (b); LSV polarization curve at different speeds (400–2400 rpm) (c and d) and K–L slope (e and f) of Pt–OCoOH and Pt.

The polarization curves of Pt–OCoOH and Pt for ORR were obtained with a rotation rate of 1600 rpm at room temperature (shown in Fig. 5a). The electrolyte was saturated by O_2_ in 0.1 M KOH, and the scan rate of the applied voltage was 10 mV s^−1^ for the performance test. The half-wave potential of Pt–OCoOH was 0.891 V, 23 mV higher than the value of the nano-particle Pt (0.868 V). The initial potential for Pt–OCoOH, shown in the polarization curve, is 1.076 V, 85 mV higher than the value of the nano-particle Pt (0.991 V). It was found that there was a large potential shift in the limiting current in Fig. 5a. Generally, the ORR limiting current is determined by the electrode surface area, the kinematic viscosity of the electrolyte, and the concentration and diffusion coefficient of the reactants. Because Pt/C and Pt–OCoOH/C have a similar value of ECSA, the electrolyte is unlikely to be different for both catalysts; the improvement in the limiting current of Pt–OCoOH/C was due to the stripping absorption rate of O species. As differences in the binding energies of oxygen species on the surface of the two kinds of catalysts are found based on theoretical calculations, the mechanism of ORR has been proved. The current density obtained after the mass normalization of noble metal Pt is called mass ratio activity. It is calculated by using formula (6); the mass activity on Pt–OCoOH is 217.88 mA mg^−1^ Pt at 0.9 V using a reversible hydrogen electrode (RHE) as the reference, and it is about 3.6 times that of the Pt nano-particle.

The ORR on the cathode catalyst is widely regarded as a multi-electron transfer process.^36^ To determine the electron transfer number on Pt–OCoOH and Pt as cathode catalysts, the polarization curves for ORR were investigated by rotating disk electrode (RDE) measurements at rotation rates from 400 to 2500 rpm in 0.1 M KOH solution (Fig. 5c and e), and the Koutecky–Levich (K–L) equations were employed to calculate the electron transfer number of ORR on the catalysts. The K–L plots for Pt–OCoOH calculated from the K–L equation at the potential of 0.5 and 0.7 V are shown in Fig. 5d (as compared with Pt/C in Fig. 5e). Based on the calculated results, the electron transfer number on Pt–OCoOH is about 3.97, which indicated that the ORR on Pt–OCoOH was a 4-electron process, same as that on the Pt electrode.^55^

**Fig. 6:**
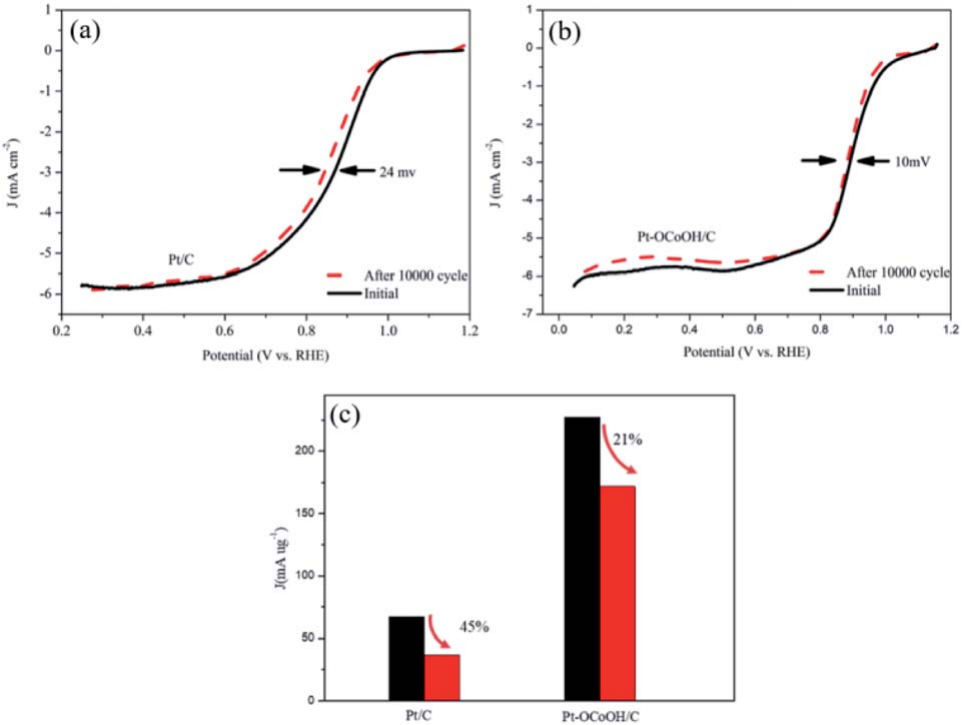
Half-wave potential attenuation of Pt/C (a) and Pt–OCoOH/C (b) catalysts after 10 000 cycles with accelerated durability test. (c) Mass ratio losses for the activity of Pt/C and Pt–OCoOH/C catalysts after 10 000 cycles.

As shown in Fig. 6a and b, the half-wave potential (*E*_1/2_) for Pt–OCoOH decreased only by 10 mV after the accelerated durability test (ADT) compared with that by 24 mV for Pt. After 10 000 cycles, the mass specific activity of Pt/C decreased by about 49%, while the activity loss of Pt–OCoOH structure was only 21% (Fig. 6c), proving the results of chemical and electrocatalytic stability of Pt–OCoOH. It means that the as-prepared Pt–OCoOH has higher stability for ORR than as-prepared Pt catalysts, due to the strong interaction between nano-islands of –OCoOH and the Pt layer.

### Theoretical calculation

**Fig. 7:**
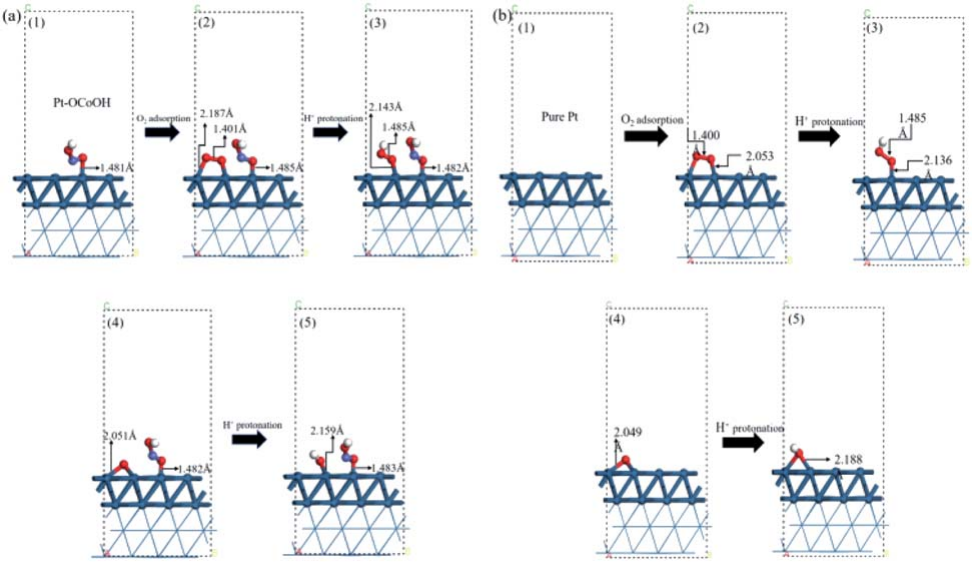
The atomic structure of the optimization process on (a) Pt–OCoOH and (b) Pt (dark blue, light blue, red and white represent Pt, Co, O and H atoms, respectively).

To understand how the introduced group enhances the ORR activity, the energetics and chemical bond length for oxygen reduction reaction on the surface of Pt–OCoOH were obtained by calculation based on DFT. Compared with pure Pt, the changes in the length of constituent bonds before and after oxygen adsorption on Pt–OCoOH are presented in Fig. 7a and b.

When O_2_ is adsorbed and protonated to form *OOH, the O–O bond in Pt–OCoOH is elongated (from 1.401 Å to 1.485 Å) following the same trend as that on the pure Pt surface. The bond lengths of O–O* (* represent the adsorption site is Pt) before and after protonation are highly consistent with those on the pure Pt (111) surface (1.401 Å, 1.485 Å for Pt–OCoOH; 1.400 Å, 1.485 Å for Pt).

The elongation of the O–O* bond and the shortening of the Pt–O bond formed by the adsorption of oxygen onto the Pt atom in the adjacent Pt–OCoOH means that the exposed O atom is more easily dissociated by protonation. After protonation, the length of the Pt–OH bond was 2.159 Å on Pt–OCoOH and 2.188 Å on Pt (111). Finally, the length of the Pt–O bond in Pt–OCoOH has no significant difference during the ORR reaction, and it indicated that the structure of Pt–OCoOH had high chemical stability.

**Fig. 8:**
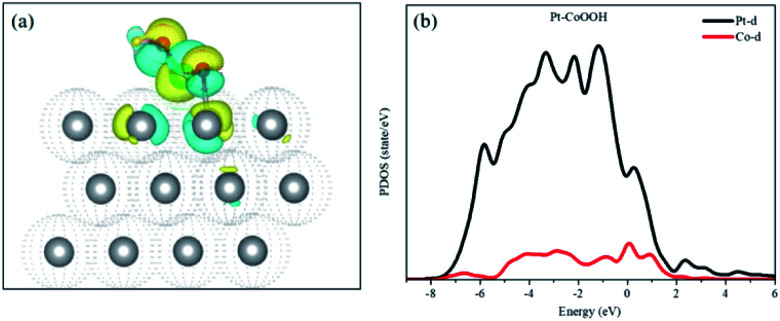
Isostructural charge density difference plots of Pt–CoOOH (a) the iso-surface level is *δ* = 0.005 e Å^−3^. Yellow and blue parts represent charge depletion and accumulation, respectively; projected density of states of OCoOH on Pt supports (b) black and red lines denote Pt d-orbital and Co d-orbital, respectively.

Bader charge and isostructural charge density difference were calculated demonstrating the electronic interactions between the introduced groups and the support of the Pt layer and Pt–OCoOH. As shown in Fig. 8(a), the transition metal Co atom bonded with hydroxide is connected to the Pt atom with the stable covalent bridge oxygen bond. The charge difference results show that the electrons and orbital are attributed to Pt atoms and bridging oxygen. Previous studies have also found that the strength of the bridged oxygen bond depended on the number of electrons provided by the precious metal. The more electrons Pt contributes, the more stable the bridged oxygen bond.^31^ Due to the introduction of the –OCoOH group, the electron cloud density on the surface of the adjacent Pt decreases (transfer 0.4e to –OCoOH). The predicted density of state (PDOS) of Pt–OCoOH (Fig. 8b) illustrated that the d-band center of Pt was away from the Fermi energy level, and the value of the d-band center was 2.88 eV calculated by formula (2), and the negative shift was 0.15 eV, compared with the d band center of the original pure Pt. According to the relation between the d-band center and chemical adsorption,^56,57^ the negative shift of the d-band center is beneficial to the adsorption capacity of Pt for oxygen.

Subsequently, the adsorption energies of five oxygen-containing species (O_2_, OOH^−^, O, OH and H_2_O) on the surfaces of pure Pt and Pt–OCoOH were respectively calculated. The adsorption energies of Pt–OCoOH and pure Pt on different surfaces are shown in Table 2. It is obvious that different surfaces can give different *E*_ads_ values for the same species. Compared with pure Pt, the adsorption energies of the O species on the surface of Pt–OCoOH all decreased (0.05–0.3 eV). It was caused by the negative shift of the center of the d-band of Pt with the introduction of the transition metal, which reduced the adsorption energies of the O species and are consistent with the density of states results. In addition, the most stable adsorption structure appeared on the adsorbed O atoms, with Pt giving 3.80 eV and Pt–OCoOH giving 3.68 eV. It is generally believed that an overstable Pt–O bond constrains the ORR rate, and the introduction of –OCoOH effectively reduces the adsorption capacity of Pt for O_2_. However, the reduction of the adsorption capacity of Pt for O_2_ was associated with the beneficial ability of dissociation of Pt–O bond by protonation.

**Table 2:** The values of the adsorption energy (*E*_ads_ (eV)) of the ORR species on Pt (111) and Pt–OCoOH

	*E*_ads_ (eV)		Adsorption energy difference
	Pt–OcoOH	Pt (111)	
O_2_	−0.761	−0.817	−0.056
OOH	−0.938	−0.943	−0.005
O	−3.457	−3.818	−0.361
OH	−1.697	−2.126	−0.429
H_2_O	−0.285	−0.32	−0.035

**Fig. 9:**
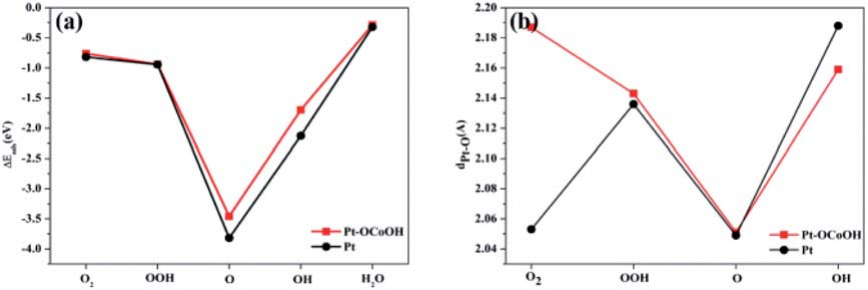
Comparison with (a) surface adsorption energy and (b) Pt–O bond length of different oxygen species.

The capacity of desorption was described by the bond length of Pt–O, that is to say, the longer the bond length of Pt–O bond, the easier the desorption, and the smaller the absolute value of the corresponding adsorption energy. Compared with pure Pt, Pt atom adjacent to Pt–OCoOH has a longer Pt–O bond after the oxygen species adsorption reaction, corresponding to a weaker adsorption capacity. However, the adsorption energy and the length of the Pt–OH* bond showed crosscurrent (Fig. 9a and b), which may be due to the introduction of the same functional group OH in –OCoOH that reduces the adsorption capacity of Pt–OH.

## Conclusions

As characterized by HRTEM and XPS, –OCoOH were proved to be highly dispersed and adsorbed onto the Pt layer as the nano-islands *via* the Pt–O chemical bond. The electrocatalytic performance and stability test indicated that Pt–OCoOH is highly stable and active compared with pure Pt. The results of DFT calculation also prove that the highly dispersed introduction of –OCoOH groups changes the d-band center of Pt, reduces the adsorption capacity for O species, and enhances the activity of ORR. The oriented introduction of the Co hydroxylated structure on the surface of Pt is not only conducive to the activity improvement of the ORR, attributing to the intensified dissociation of oxygen by protonation and reduced chemical adsorption of oxygen species, but also enhances the chemical stability of the electron materials by preferential hydroxylation.

## Conflicts of interest

There are no conflicts to declare.

## Supplementary Material

RA-010-D0RA08645B-s001
