# Supplementary material for: The mechanism of Co oxyhydroxide nano-islands deposited on a Pt surface to promote the oxygen reduction reaction at the cathode of fuel cells
Source: RSC Adv. 2020 Dec 17;10(73):44719–27. doi: 10.1039/d0ra08645b (PMC9058475; doi:10.1039/d0ra08645b)
Supplement: RA-010-D0RA08645B-s001 [file RA-010-D0RA08645B-s001.pdf]

Electronic Supplementary Material (ESI) for RSC Advances.
This journal is © The Royal Society of Chemistry 2020

SUPPLEMENTARY INFORMATION

The Enhancement Mechanism of Co Oxyhydroxide groups Deposited on Pt Surface for Oxygen Reduction Reaction with Cathode in Fuel Cell

Jinghao Lu[a], Libin Yang[a,], Wei Guo[a], Songtao Xiao[b], Lingyu Wang[b], Yinggen OuYang[b], Peng Gao[a]*

[a]College of Chemical Engineering and Materials Science/Tianjin Key Laboratory of Brine Chemical Engineering and Resource Eco-utilization, Tianjin University of Science and Technology. E-mail: yanglibin@tust.edu.cn (Y.L); Tel.:86-13752339079 (Y.LB)

[b]Department of Radiochemistry, China Institute of Atomic Energy, Beijing 102413, China

*Corresponding author: Libin Yang(Email: yanglibin@tust.edu.cn)

Reference	Product	Ink				Amount of ink coating (ul)	Working electrode size (mm)	Electrolyte solution	Limiting current density (mA/cm^2)	Pt loading (ug/cm^2)
		Catalyst mass (mg)	Solvent volume	Water volume (ml)	Nafion (ul)					
1	PtPb/vxc-72	1	0	1	15	20	5	0.1M $HClO_4$	5.6	15.3
2	PtNi /vxc-72	2	1ml methanol	0	none	10	3	0.1M $HClO_4$	5.8	28
3	Pt_3Co /vxc-72	2	0.2ml Isopropyl alcohol	0.8	20	10	5	0.1M HClO	6	20
4	L_1-PtCo /vxc-72	2	0.9ml Isopropyl alcohol	0	100	10	5	0.1M HClO	5.8	20
5	$Pt_{75}Co_{25}$	1	1ml ethanol	0	4	6	3	0.5M KOH	6	40
6	$Pt_{78}Co_{22}$ NCs/rGO	1	none	1	4	3	3	0.5M KOH	6	20
8	PtCoNi /vxc-72	1	1ml ethanol	0	40	3	3	0.5M KOH	6	18
This work	Pt/vxc-72	2	0.5ml ethanol	0.5	20	3	3	0.1M KOH	5.6	16
This work	Pt-OCoOH /vxc-72	2	0.5ml ethanol	0.5	20	3	3	0.1M KOH	5.6	14.4

Table S1 Summary of ink coating amount, catalyst amount and Pt loading amount.
